# Exploring Free Energies of Specific Protein Conformations Using the Martini Force Field

**DOI:** 10.1021/acs.jctc.3c01155

**Published:** 2024-03-01

**Authors:** Wojciech Plazinski, Valery Lutsyk, Anita Plazinska

**Affiliations:** † Jerzy Haber Institute of Catalysis and Surface Chemistry, Polish Academy of Sciences, Niezapominajek 8, Krakow 30-239, Poland; ‡ Department of Biopharmacy, Medical University of Lublin, Chodzki 4a, Lublin 20-093, Poland

## Abstract

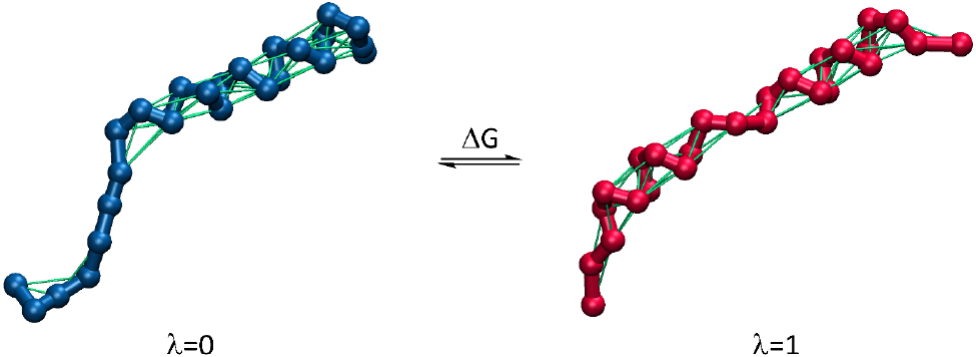

Coarse-grained (CG) level molecular dynamics simulations are routinely used to study various biomolecular processes. The Martini force field is currently the most widely adopted parameter set for such simulations. The functional form of this and several other CG force fields enforces secondary protein structure support by employing a variety of harmonic potentials or restraints that favor the protein’s native conformation. We propose a straightforward method to calculate the energetic consequences of transitions between predefined conformational states in systems in which multiple factors can affect protein conformational equilibria. This method is designed for use within the Martini force field and involves imposing conformational transitions by linking a Martini-inherent elastic network to the coupling parameter λ. We demonstrate the applicability of our method using the example of five biomolecular systems that undergo experimentally characterized conformational transitions between well-defined structures (*Staphylococcal* nuclease, C-terminal segment of surfactant protein B, LAH4 peptide, and β_2_-adrenergic receptor) as well as between folded and unfolded states (GCN4 leucine zipper protein). The results show that the relative free energy changes associated with protein conformational transitions, which are affected by various factors, such as pH, mutations, solvent, and lipid membrane composition, are correctly reproduced. The proposed method may be a valuable tool for understanding how different conditions and modifications affect conformational equilibria in proteins.

## Introduction

Molecular dynamics (MD) simulations carried out at the coarse-grained (CG) resolution level have diverse applications in exploring processes related to biomolecules.^1−4^ The accuracy and quality of simulation outcomes and resulting conclusions rely significantly on the type of force field used.^5,6^ Currently, the most popular force field in the CG family is Martini.^7^ Its latest version (v. 3) includes mutually compatible parameters for proteins, lipids, phospholipids,^8−10^ carbohydrates,^11,12^ solvents,^8^ and a number of other organic compounds.^13^

In the context of proteins, one of the major limitations of Martini is the restriction of conformational freedom by a number of force field parameters that depend on the 3D structure of a given protein. Secondary and tertiary structures are imposed by additional force field terms, following approaches such as elastic networks.^14^ The defined network of restraints (elastic network) aims to maintain the structural and dynamical properties of a protein, including its collective motions, comparable to those obtained using atomistic models.^14^ The set of restraints is based on harmonic potentials, where the minima correspond to distances between selected CG beads according to the structures on which the model is based (typically structures from the PDB database). Pairs of CG beads for which restraints are defined are chosen based on the criterion of their distances, with typical cutoff values ranging from 0.7 to 1.0 nm, while recommended force constants vary in the range of 500–1000 kJ/mol/nm^2^. Although the general idea of the restraint network was introduced for Martini 2.1, it also applies to the latest Martini version (3.0), serving as the standard for describing protein structures within this force field.^7^ The alternative for elastic network approach is the Go̅-Martini model^15^ which partially mitigates the problem of excessive rigidity in the structure of the studied protein by replacing harmonic potential restraints with Lennard-Jones (LJ) potentials for selected pairs of CG beads. This facilitates the exploration of conformational states farther away from the native structure for which the set of restraints was generated. However, even in this case, the use of LJ potentials favors the native structure and biases the sampling toward native conformations over the others. Therefore, both most commonly used approaches are based on favoring one native structure of protein by adding appropriate elements (harmonic restraints or LJ potentials) to the system’s Hamiltonian. As a result, the study of large-scale conformational changes can be challenging or even impossible in the case when such a change involves significant deviation from at least one energy minimum imposed in the elastic network. In real protein systems considered in the context of their biological function, it is often necessary to consider two or more different conformers, along with the dynamic equilibrium corresponding to the conformational transition between them as well as numerous natural and other factors capable of altering this equilibrium.

In this work, a theoretical approach is presented that allows the quantitative determination of the effects of various factors on the conformational changes of biomolecules simulated in the Martini force field. The proposed approach requires knowledge of reference structures that define the limiting stages of the conformational transition under study. Its outcome results in a quantitative estimation of the energetic effect accompanying such a transition in systems that differ by selected factors. These factors can include both external conditions that affect the studied protein, such as temperature, solvent composition, pH, lipid membrane composition (in the case of membrane proteins), and presence of ligands, as well as changes in the protein’s own character, such as point mutations. The described method is based on standard coarse-grained MD simulation protocols combined with the use of coupling parameters describing the transition from one well-defined conformational state to another and subsequent free energy calculations.

The general idea of using free energy calculations for two predefined conformational states is not new and encompasses many well-grounded approaches in the field of biomolecular simulations, including, among others, targeted MD (conformational change is driven by holonomic constraints on the root-mean-square deviation between the current and target structures),^16−19^ steered MD (a harmonic restraint based on a reference point moves the system toward the target as the reference point is updated),^20,21^ biased MD (the system feels no force as it moves toward the target, and the bias potential is nonzero only as the system moves away from the target),^22,23^ or umbrella sampling (a single one of a series of biased MD simulations with the purpose of reweighting the data to obtain a thermodynamically correct average of the free energy profile).^24^ These methods have in common that the transition between two end states is controlled by a progress variable (reaction coordinate), although they differ in the way the progress variable is controlled.^25^ The focused confinement method^26,27^ also uses the perturbation formalism, but at certain intermediate stages of the calculation, and the desired quantity, i.e., the free energy change associated with the conformational transition, is assumed to be coordinate independent.

Methodologically, the current approach is closest to the method proposed in ref (28) due to the fact that in both cases, perturbed distance restraints are used, and multiple distance restraints are coupled to a single parameter λ. In the current case, the main difference lies in the interference with parameters that are directly responsible for describing the higher-order structure of the protein (in this case, the elastic network of distance-based restraints inherent to the Martini family of force fields). This does not apply to the case of atomistic force fields, where the system’s Hamiltonian is, in general, independent of the structure of the considered biomolecule. Moreover, since the use of an elastic network is standard for the Martini force field, applying a method relying on perturbed restraints provides the opportunity to automate the entire procedure and its use for a quantitative description of the thermodynamic characteristics for the general case of conformational changes in any protein.

The applicability of the proposed method is illustrated by five biomolecular systems (*Staphylococcal* nuclease, C-terminal segment of surfactant protein B, LAH4 peptide, GCN4 leucine zipper protein, and human β_2_-adrenergic receptor), for which well-defined conformational transitions influenced by a variety of factors have been experimentally confirmed and structurally characterized.

## Theory

Let us begin by defining two different structures, A and B, of the same protein. It is assumed that their conformations are different enough to require separate sets of Martini parameters, i.e., states A and B are characterized by different sets of bonds/restraints (elastic networks or compatible) that preserve the secondary and tertiary structure of the respective conformer. On the contrary, the remaining elements of the model (e.g., parameter related to bead–bead bonds not included in the elastic network, regular and dihedral angles, nonbonded parameters, etc.) are usually the same for both A and B. A model of this type imposes distance-dependent restraints only on the protein backbone structure. CG beads belonging to protein side chains are not included in the elastic network. The elastic network-based restraints necessary to maintain the structure of the protein take the form of harmonic potentials by default, i.e., they have a single energetic minimum, and spontaneous transformation from conformer A to B (or *vice versa*) is not possible, regardless of the time scale of the simulation.

According to the proposed approach, the transformation from state A to B occurs in a manner analogous to enhanced-sampling methods that use the coupling parameter λ.^29^ This parameter describes the gradual transformation of selected force field parameters that differ between states A and B. Consequently, state A corresponds to a value of λ = 0, and state B corresponds to λ = 1, while all intermediate states with values in the range 0 < λ < 1 correspond to structures along the transformation path from A to B. Then, the free energy change associated with such a transformation can be calculated from ensemble-averaged enthalpy changes along the A-to-B path (Δ*G*_AB_) by using, e.g., thermodynamic integration^30^ or Bennett acceptance ratio^31^ methods. Noting that the Δ*G*_AB_ value is dependent on the set of conditions associated with both the system and the simulation protocols, we denote this set as **X** and the corresponding free energy change as Δ*G*_AB_(**X**).

Due to the fact that all perturbed terms of the force field have the character of a harmonic potential (*V*_h_), their dependence on the value of the λ parameter can be described by eq 1
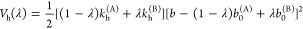
1where *k*_h_ are force constants for harmonic potentials present in states A and B and *b*_0_ are minima of these potentials. The above equation includes all of the force field elements that depend on the value of the λ parameter. In some cases, states A and B differ in the value of the parameter *b*_0_, while maintaining the same value of *k*_h_; there may also be a situation where one of the limit values of *k*_h_ (i.e., in either state A or B) is equal to zero, corresponding to the absence of any harmonic potential. A special case, in the context of the considered systems, is the dependence of both nonzero *k*_h_ and *b*_0_ on λ, as is the case for the β_2_-adrenergic receptor. Due to differences in the 3D structures between the A and B states and the absence of certain receptor structural elements (loops and termini), some bead–bead interactions are automatically assigned to the type of bonds or to an elastic network. Regardless of this technical aspect, the functional form of such interactions remains the same (eq 1).

The perturbation designed in this way includes only the elements constituting the elastic network, while excluding any other elements of the force field (with small exceptions resulting from the automatic assignment of certain harmonic potentials to the ‘bonds’ type). However, since the elastic network itself is characteristic of the Martini force field and essential for describing the 3D structure of proteins, one can characterize this approach as equivalent to the dual topology approach, with the caveat that the only λ-perturbed elements are the mentioned restraints. Due to the fact that elastic network restraints are an integral part of the model, they are treated as an inherent component of the perturbed topology.

**Figure 1:**
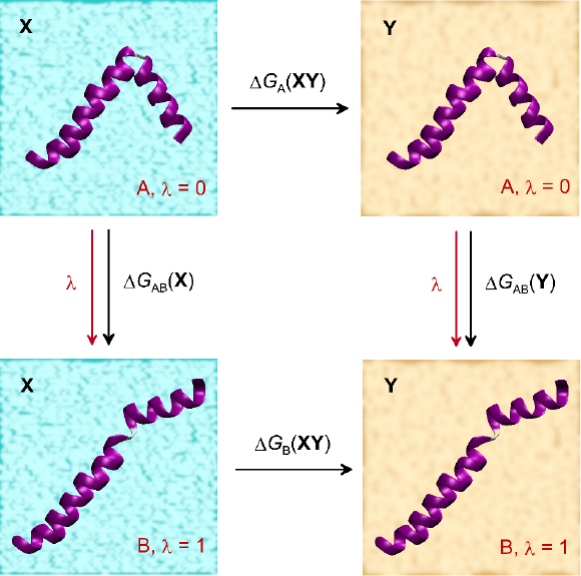
Example of a thermodynamic cycle that can be used to calculate the values of the free energy change associated with the A → B conformational rearrangement of the protein molecule induced by changing the conditions **X** → **Y**. Here, **X** and **Y** are represented by different types of solvents, and the crucial series of MD simulations are indicated by red arrows.

The calculated change in free energy is not physically meaningful as it includes not only changes in structure but also correlated changes in force field parameters. The obtained value can be compared to the value obtained from a single branch of a thermodynamic cycle corresponding to the alchemical transformation.^32^ However, the value of Δ*G*_AB_(**X**) can be useful when compared to a similar value obtained for the same protein in the same manner, but for a system that differs by one or more factors, altering the conditions of simulations from **X** to **Y**. The relative change in free energy
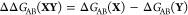
2shows how the factor (**Y**) has affected the position of dynamic equilibrium for the A → B transformation with respect to the reference system characterized by the conditions **X**. Furthermore, through the construction of a comprehensive thermodynamic cycle (Figure 1), it is possible to determine the absolute changes in free energy resulting from the A → B transformation under various conditions. This can be achieved by employing the following equation:
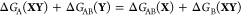
3where Δ*G*_A_(**XY**) and Δ*G*_B_(**XY**) are the values of the free energy change associated with the changes in the conditions for the fixed conformation of the protein. In such cases, it is important to have the ability to transform the set of conditions experienced by the system (**X** → **Y**) while keeping the conformation (A or B) unchanged. However, this may not always be possible, especially when performing complex transformations that require large changes in the composition of the system (e.g., a complete solvent exchange), which may lead to artifacts. In this study, we will focus on examining the relative changes in free energy, ΔΔ*G*_AB_(**XY**), induced by factors of different characteristics.

## Methods

The systems studied at the CG level are given in Table 1. They include five different proteins simulated under different conditions and exhibiting two different conformational states: *Staphylococcal* nuclease (SNase), C-terminal segment of surfactant protein B (SPB), LAH4 peptide (LAH4), GCN4 leucine zipper protein (GCN4), and the human β_2_-adrenergic receptor (β_2_-AR). The initial protein structures rely on the PDB data. Only structures labeled A states were considered to initiate the MD simulations; while B states were considered only as end points of the λ-based MD simulations and to generate the B-state parameters required to define the A → B path. The exception is the most complex protein and the most structurally significant conformational rearrangement, i.e., β_2_-AR for which the reverse transition (B → A) was additionally considered to estimate the existence of potential hysteresis. The assignment of the given PDB structure to either the A or B state is given in Table 1. The GCN4 protein lacks the structure corresponding to the B state (unfolded protein); therefore, the model for this state was prepared on the basis of the A state model by removing all the restraints involved in the elastic network supporting the secondary structure. All the structures were cleaned, i.e., all nonprotein molecules (e.g., cocrystallized ligands or water molecules), copies of the same protein structures contained in the PDB file, and (in the case of transmembrane proteins) artificially added modifications that facilitate their crystallization were removed to leave only the protein of interest. Additionally, in the case of β_2_-AR, the sizes of the protein were adjusted by removing residues at the selected loops as well as at the N- and C-termini that did not have counterparts in the second PDB input. The *insane* tool was used to solvate the considered solute molecule and to construct the initial configuration of the lipid bilayer wherever necessary. The parameters related to proteins, phospholipids (1-palmitoyl-2-oleoyl-*sn*-glycero-3-phosphocholine, POPC; 1-palmitoyl-2-oleoyl-*sn*-glycero-3-phosphoethanolamine, POPE, and 1,2-dipalmitoyl-*sn*-glycero-3-phosphocholine, DPPC), as well as solvents and ions, corresponded to the Martini 3 force field^8^ and, in the case of proteins, were generated by using the web-available tool *martinize2* by using default options with some subsequent modifications described below. The most relevant information in this context is that the default force constant value used within the elastic network is 500 kJ/mol/nm^2^ while the cutoff for generating the bead pairs involved in the elastic network is 0.9 nm. The parameters for two functionally different ligands of β_2_-AR (full agonist, epinephrine, and antagonist, propranolol) were developed on the basis of all-atom MD simulations performed in the CHARMM force field,^33−35^ according to the protocol described in other work.^13^ The CG parametrization procedure followed the Martini rules and involved adjusting the bonded parameters against the all-atom data, while the choice of the nonbonded parameters depended on the chemical properties of the groups mapped to CG beads. In particular, the nonbonded parameters from ref (10) were used at initial stages of ligand parametrization. In the case of both ligands, their amine moieties were protonated.

**Table 1:** Properties of the Simulated Systems

protein	conformational state: PDB code	box size^a^	solvent	system composition^b^	comments
SNase	A: 1KDA	8.2 × 8.2 × 8.2 nm^3^	water	4432 W + 45 Na^+^ + 52 Cl^–^	Asp116
	A: 1KDA				Glu116
	B: 1KDB				Asp116
	B: 1KDB				Glu116
SPB^c^	A: 1RG3:1	7.7 × 7.7 × 7.7 nm^3^	hexane	2418 HEX	solvent change
	A: 1RG3:1	6.8 × 6.8 × 6.8 nm^3^	2,2,2-trifluoroethanol	2418 TFEOL	
	B: 1RG4:1	7.7 × 7.7 × 7.7 nm^3^	hexane	2418 HEX	
	B: 1RG4:1	6.8 × 6.8 × 6.8 nm^3^	2,2,2-trifluoroethanol	2418 TFEOL	
LAH4	A: 2KJN:2	8.3 × 8.3 × 8.2 nm^3^	water	4691 W + 44 Na^+^ + 52 Cl^–^	His10^+^, His11^+^, His14^+^, His18^+^^d^
	A: 2KJN:2			4691 W + 46 Na^+^ + 52 Cl^–^	His10^+^, His11^+^, His14°, His18°^d^
	A: 2KJN:2			4691 W + 48 Na^+^ + 52 Cl^–^	His10°, His11°, His14°, His18°^d^
	A: 2KJN:2			4691 W + 44 Na^+^ + 52 Cl^–^	His10^+^, His11°, His14°, His18^+^^d^
	B: 2KJO:1			4691 W + 44 Na^+^ + 52 Cl^–^	His10^+^, His11^+^, His14^+^, His18^+^^d^
	B: 2KJO:1			4691 W + 46 Na^+^ + 52 Cl^–^	His10^+^, His11^+^, His14°, His18°^d^
	B: 2KJO:1			4691 W + 48 Na^+^ + 52 Cl^–^	His10°, His11°, His14°, His18°^d^
	B: 2KJO:1			4691 W + 44 Na^+^ + 52 Cl^–^	His10^+^, His11°, His14°, His18^+^^d^
GCN4	A: 2ZTA	8.3 × 8.3 × 8.3 nm^3^	water	4660 W + 51 Na^+^ + 51 Cl^–^	Arg25
	A: 2ZTA				Ala25
	B: -				Arg25
	B: -				Ala25
β_2_-AR	A: 2RH1	13.8 × 13.8 × 13.6 nm^3^	water	14 922 W + 607 DPPC + 159 Na^+^ + 166 Cl^–^	*Apo*, various membranes
	A: 2RH1	14.5 × 14.5 × 12.5 nm^3^		14 922 W + 607 POPC + 159 Na^+^ + 166 Cl^–^	
	A: 2RH1	14 × 14 × 13 nm^3^		14 922 W + 607 POPE + 159 Na^+^ + 166 Cl^–^	
	A: 2RH1	13.8 × 13.8 × 13.6 nm^3^		14 866 W + 607 DPPC + 159 Na^+^ + 166 Cl^–^ + 10 ADR	bound ligand, same membrane
	A: 2RH1	13.8 × 13.8 × 13.6 nm^3^		14 866 W + 607 DPPC + 159 Na^+^ + 166 Cl^–^ + 10 PROP	
	B: 3P0G	13.8 × 13.8 × 13.6 nm^3^		14 922 W + 607 DPPC + 159 Na^+^ + 166 Cl^–^	*Apo*, various membranes
	B: 3P0G	14.5 × 14.5 × 12.5 nm^3^		14 922 W + 607 POPC + 159 Na^+^ + 166 Cl^–^	
	B: 3P0G	14 × 14 × 13 nm^3^		14 922 W + 607 POPE + 159 Na^+^ + 166 Cl^–^	
	B: 3P0G	13.8 × 13.8 × 13.6 nm^3^		14 866 W + 607 DPPC + 159 Na^+^ + 166 Cl^–^ + 10 ADR	bound ligand, same membrane
	B: 3P0G	13.8 × 13.8 × 13.6 nm^3^		14 866 W + 607 DPPC + 149 Na^+^ + 166 Cl^–^ + 10 PROP	

a After equilibration.

b Not including protein. W = Martini water; HEX = hexane; TFEOL = 2,2,2-trifluoroethanol; PROP = propranolol; ADR = epinephrine.

c Considered in the context of three different values of force constants for restraints defining the elastic network (see the Methods section).

d His residues can have either a positive charge (+) or be neutral (0).

In selected proteins, some of the parameters responsible for supporting the secondary and tertiary structure but assigned to disordered fragments of these proteins have been removed. These alterations impact the intra- and extracellular loops of β_2_-AR and the flexible parts of LAH4 and SPB. The flexible nature of the fragments affected by these modifications can be seen by examining the structural data for these proteins in the PDB database. To allow the transition from state A to state B during the simulations, the relevant parameters defining the elastic network responsible for maintaining the secondary and tertiary structures of the protein were modified. This modification was in accordance with the dual-topology approach used in each simulation with a λ coupling parameter. For all the systems considered, the modifications introduced had the greatest importance in the’Rubber band’ section (within the [bonds] directive in the GROMACS topology file), which was automatically generated by the *martinize2* program based on the protein structure. A handwritten *bash* script was used to mix the A and B state parameters in this section; the script is provided together with the final topology files in the Supporting Information. Remaining parameters that differed between the *martinize2*-generated topologies for states A and B were modified manually. This includes, e.g., protonation states or perturbations within the [bonds] directive outside the ‘Rubber bands’ section (only for the case of β_2_-AR). Moreover, in order to enable the λ-based MD simulations in GROMACS, all the ‘restricted angle’ potentials (GROMACS type 10) have been converted to regular angles (GROMACS type 2) while keeping the same parameters of the corresponding functions.

Additionally, to examine how the force constant value in the perturbed restraint network affects the final results, additional simulation series were conducted for the SPB protein with modified values of the aforementioned constants. Specifically, twice smaller and twice larger values were used for all restraints in the elastic network while keeping the other simulation and model parameters unchanged. It should be noted that only one of these cases (twice larger force constant values) falls within the recommended range, allowing for realistic behavior of the CG protein structure (according to ref (14) ).

MD simulations were carried out with the GROMACS 2023.2 package,^36^ with periodic boundary conditions and in the isothermal–isobaric ensemble. The system temperature was maintained near the reference value of 310 K using the V-rescale thermostat,^37^ while constant pressure (1 bar) was regulated using the Parrinello–Rahman barostat^38^ with a relaxation time of 40 ps. The pressure scaling was either semi-isotropic (bilayer systems) or isotropic (remaining systems). The equations of motion were integrated with a time step of 5 fs using the leapfrog scheme.^39^ At each time step, the translational center-of-mass motion was removed separately for the solute, solvent, and bilayer (if present). The Lennard-Jones potentials for van der Waals interactions were shifted to zero beyond a cutoff distance of 1.1 nm. For Coulomb interactions, the reaction-field approach was used with a cutoff of 1.1 nm and ε_*r*_ = 15, 2, and 9 (for water, hexane, and trifluoroethanol, respectively). Other MD parameters were maintained in accordance with the example *mdp* files that have been deposited on the *cgmartini.nl* web site.

Equilibration simulations were performed for a duration of 100 ns. After this stage, production simulations were started from the last frame of the equilibration trajectories for all systems except those containing ligand-bound β_2_-AR. For systems containing both β_2_-AR and its ligands, the equilibration state was extended to the point where one of the ligand molecules was fully bound in the receptor cavity. Then, a restraint was imposed on the distance between the charged bead of the ligand molecule (representing amine moiety) and the bead of the Asp113 side chain to prevent the spontaneous dissociation of the ligand molecule from the cavity. The restraint had a character of the *upper wall* potential implemented in PLUMED 2.6,^40^ with a force constant equal to 2500 kJ/mol/nm^2^ and a wall position of 0.4 nm. After further equilibration of 100 ns, such ligand-bound systems were passed to production simulations.

The MD simulations involved a gradual conformational transition from state A to B (see Table 1) in a stepwise manner as a function of coupling parameter λ. The associated free energy changes were calculated using the Bennett acceptance ratio (BAR) method^31^ implemented in the GROMACS *gmx bar* subroutine. This included the error estimate determined using the default GROMACS criteria, i.e., by dividing the data into blocks, determining the free energy differences over these blocks, and assuming that the blocks are independent. The final error estimate was determined from the average variance over 5 blocks according to the default criteria implemented in the gmx bar. The dependence of the estimated error on the number of blocks (varying from 2 to 500 000) was also examined to confirm that the number of blocks less than 10 results in a nearly constant value of the estimated error.^41^ The 29 λ-points were accepted (λ = 0, 0.0167, 0.033, 0.05, 0.075, 0.1, 0.125, 0.15, 0.2, ..., 0.8, 0.85, 0.875, 0.9, 0.925, 0.95, 0.967, 0.983, 1), and the data of the equilibrated systems were collected every 50 ps for a duration varying from 2.4 to 8 μs in each λ window until convergence was reached. For some test simulations aimed at determining the effect of the inverted direction of the transition (B → A vs. A → B), a smaller number of evenly distributed λ-points (21) were used. The convergence of the Δ*G*_AB_ values was validated using hand-written scripts.

## Results and Discussion

### General Characteristics

**Figure 2:**
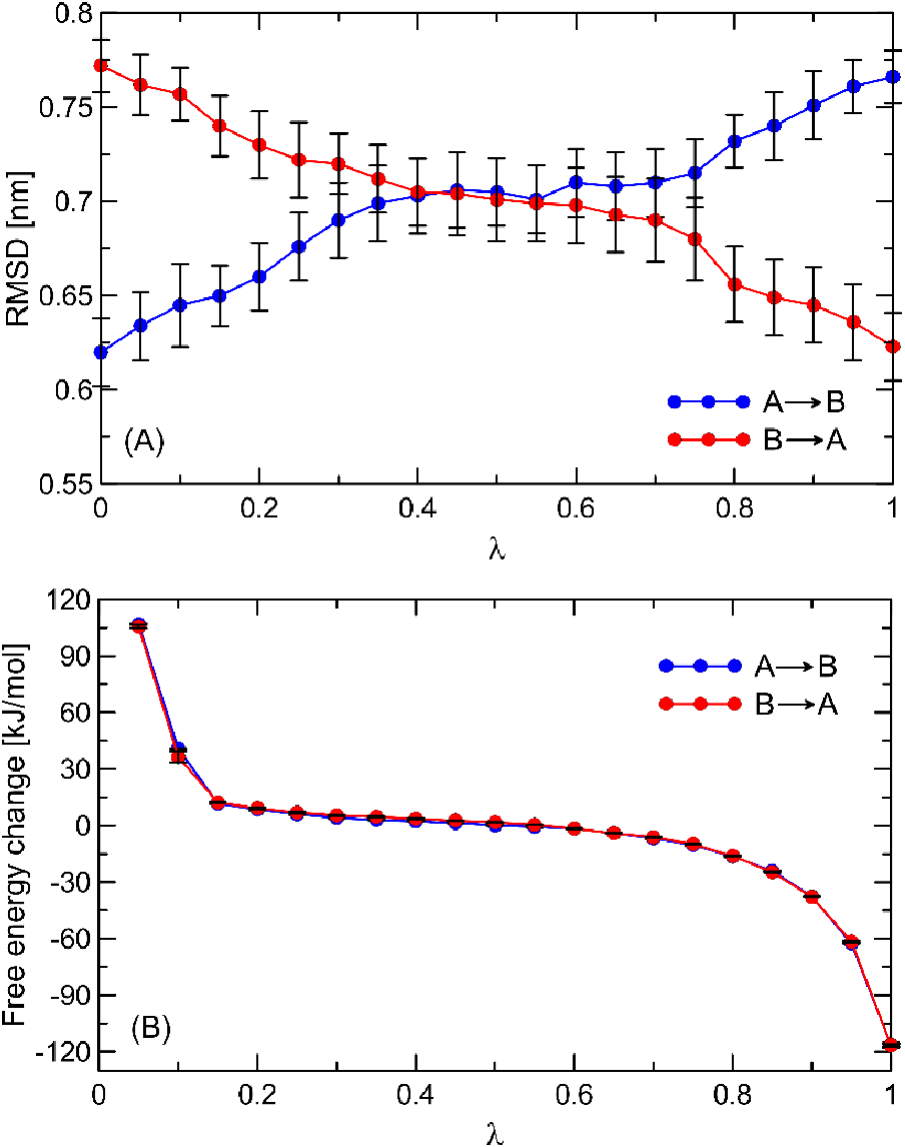
Structural and energetic changes experienced by β_2_-AR during changes of the λ parameter. (A) The average root-mean-square deviation (RMSD) values for the entire MD trajectory with respect to state A’s equilibrated structure. The vertical bars correspond to 20% of the standard deviations. (B) The partial energy changes were determined for each intermediate λ point by using the BAR method. The vertical bars represent the BAR-estimated errors.

For all systems studied, an evolution of structures was observed, which was correlated to a gradual change in the value of the λ parameter. The end points of these migrations (states B) corresponded to the structures expected from the PDB data. For example, steric clashes trapping the system at an intermediate stage of the transformation from A to B and preventing the completion of the full pathway defined on the λ-value were not observed. Moreover, the energetic characteristics of the A → B transition proved to be fully reversible, and the same intermediate energy change values as well as the final Δ*G*_AB_ value remained in good agreement with analogous values calculated for the reverse transition, i.e., B → A. (This aspect was investigated in the context of the most complex system, i.e., β_2_-AR, for which such potential differences were most likely to occur.) The relevant results regarding this issue are presented graphically in Figure 2.

**Figure 3:**
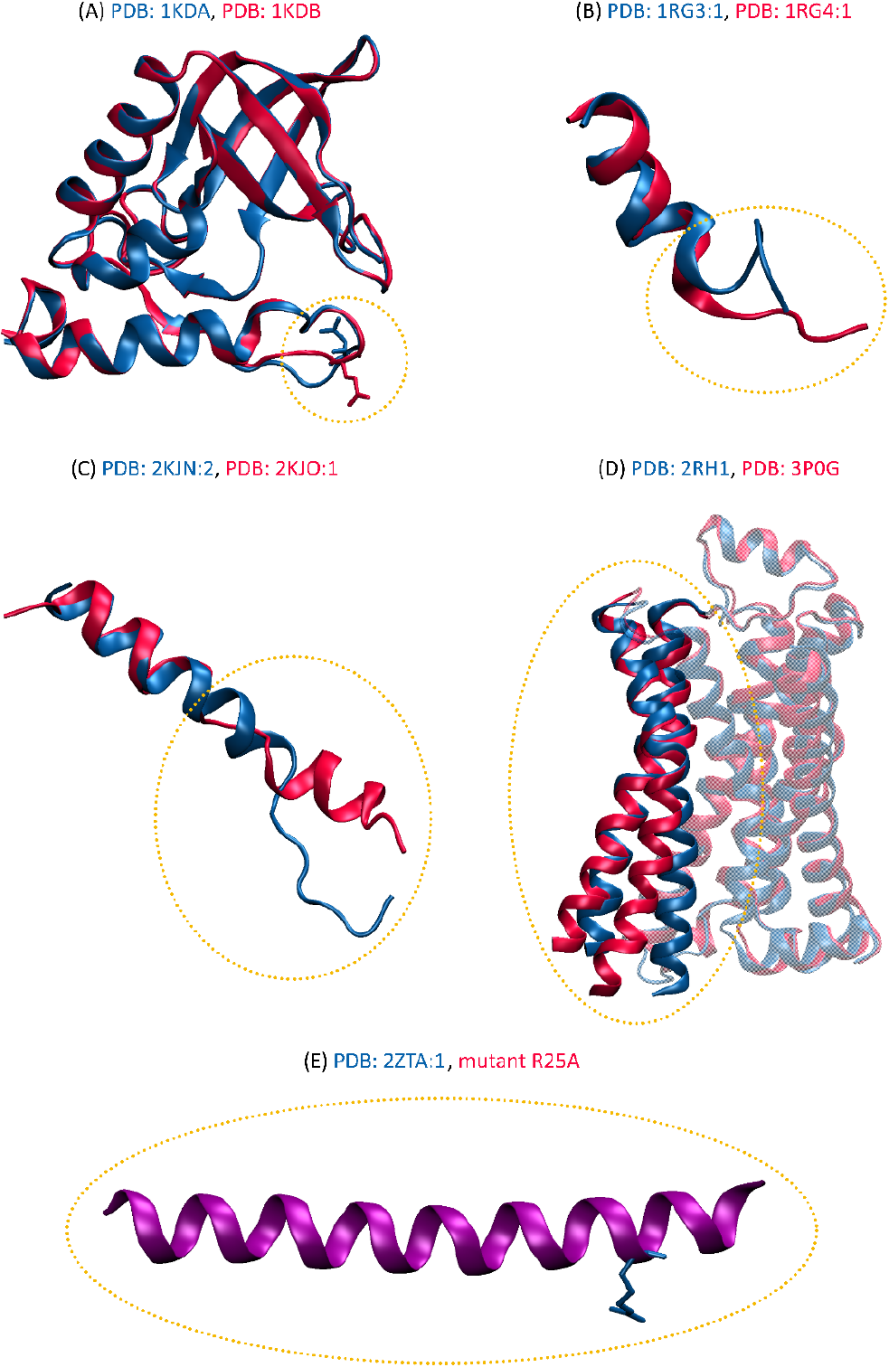
(A–D) The superposition of the two conformational states (A and B) determined by experimental studies (XRD or NMR) for the four proteins considered: (A) *Staphylococcal* nuclease, (B) C-terminal segment of surfactant protein B, (C) LAH4 peptide, and (D) the human β_2_-adrenergic receptor. The structures were superposed based on the PDB entries listed in Table 1. State A is shown in blue and state B in red. (E) The structure of the folded state A of the GCN4 protein corresponds to both wild-type protein and Arg25Ala mutant; state B corresponds to unfolded protein (not-shown).

The structural features of conformational transitions within four (out of five) molecular systems considered in this article are illustrated in Figure 3. For a detailed description, the reader is referred to the original papers, which describe both the molecular details of the conformational changes and the factors that induce them.^42−47^ The results of the free energy calculations for selected systems are summarized in Table 2 and briefly discussed in the following paragraphs. In cases where well-defined structural data are available and the factor influencing the conformational equilibrium can be accurately reproduced in the model, the reported results serve as validation, demonstrating the applicability of our procedure.

**Table 2:** Summary of Considered Systems, Factors Influencing the Conformational Equilibrium, and the Results of MD Simulations Carried Out According to the Presently Proposed Methodology

protein	factor influencing the conformational equilibrium	implementation in MD setup	experimentally inferred shift of conformational equilibrium	considered transitions	ΔΔ*G*_AB_(kJ/mol)^a^
SNase	mutation	Asp116	preferred state A	A → B^b^	0
		Glu116	preferred state B	A → B	–1.68 ± 0.26
SPB	solvent type	hexane	preferred state A	A → B^b^	0
		2,2,2-trifluoroethanol	preferred state B	A → B	–6.65 ± 0.04
LAH4	pH	all His protonated	preferred state A	A → B^b^	0
		His14 and His18 protonated	preferred state B	A → B	–7.20 ± 2.44
		all His deprotonated	closer to A	A → B	2.12 ± 2.04
		His10 and His18 protonated		A → B	1.06 ± 2.66
GCN4	mutation	Arg25	preferred state A, the Arg25Ala mutation changes folding energy by 6.95 kJ/mol	A → B^b^	0
		Ala25		A → B	5.40 ± 2.62
β_2_-AR	composition of lipid bilayer	pure DPPC		A → B^b^	0
		pure POPC		A → B	10.01 ± 5.88
		pure POPE	toward B	A → B	–2.50 ± 4.09
	presence of bound ligand	epinephrine	preferred state B	A → B	12.42 ± 5.01
		propranolol	preferred state A	A → B	12.67 ± 5.78

a Error estimated by the GROMACS-default method (see the Methods section) and summation of errors corresponding to both Δ*G*_AB_ values.

b Reference transition.

Furthermore, autocorrelation functions (ACFs) were generated for the collected ∂H/∂λ data, which form the basis for free energy calculations. Both the behavior of the ACF (rapid decay toward zero values for times up to a few tens of nanoseconds) and roughly estimated autocorrelation times (ranging from tens of picoseconds to tens of nanoseconds) confirm that data collected for times reaching several microseconds are decorrelated and can be used for calculations employing the BAR method.

In view of potential problems associated with modeling the structure of unfolded proteins (the case of the GCN4 system), in addition to collecting input data for BAR, the conformational characteristics for state B were also examined. Significant conformational variability of the unfolded protein was observed, manifested by a wide range of end-to-end distances of the peptide chain (from ca. 0.5 to 7 nm) changing on the time scale of tens of nanoseconds. This indicates the sampling of multiple disordered structures (as is characteristic of the unfolded protein) and suggests that the collected data do not correspond to only one conformationally locked structure.

### *Staphylococcal* Nuclease

As demonstrated in ref (42) a mutation at position number 116 can affect the structure of the SNase protein. The two mutations in the wild-type protein, namely, Lys116Asp and Lys116Glu, lead to a different shape of the loop within residues 112–117, resulting in conformations referred to as states A and B, respectively, in the present study. The most significant changes involve residues Tyr115 and Asp/Glu116, where the different conformations affect both the backbone and side chains. The changes in conformational free energy for the A → B transition are more favorable for the Lys116Glu mutant compared to the reference system (state A, Lys116Asp mutant), which is consistent with the data reported in the original study^42^ and the structures in PDB: 1KDA and 1KDB. The relative energy changes, ΔΔ*G*_AB_, are relatively small, equal to ca. −1.7 kJ/mol, suggesting a high mobility of the loop and its conformational accessibility to both structures, regardless of the residue type at position 116. According to this scenario, chemically similar residues Asp and Glu at position 116 primarily determine the dynamic equilibrium state, switching the loop geometry between states A and B and *vice versa*.

### LAH4 Peptide

The conformation of the LAH4 peptide was considered in the context of varying the pH, which directly affects the protonation of the histidines. According to ref (43) at a low pH of 4.1, the peptide molecule adopts a helical conformation between residues 9 and 24 (state A), whereas at a higher pH of 6.1, a helix–loop–helix structure is formed with a hinge encompassing residues His10-Ala13 (state B). At an even higher pH of 7.8, all histidines are uncharged, and an extended helical conformation is again obtained. It should be noted that the change in charge and conformation of the peptide correlates with its affinity for micelles, but the simplified model used in this case (peptide in water) aims to capture only the conformational preferences induced by the pH changes. Additionally, only structures corresponding to the two lower pH values are available, allowing only a partial description of the conformational transitions.

The A → B transition is interpreted as a result of early deprotonation of His10 and His11, which was considered in the current study as one of the conditions opposing the full protonation of LAH4 (low pH conditions). In addition to these two cases, the two further systems were considered, namely: complete deprotonation of LAH4 (high pH conditions) and an alternative pattern of protonation with protonated His10 and His18. The calculated relative changes in free energy, ΔΔ*G*_AB_ = −7.20 ± 2.44 kJ/mol, confirm that the transition from A to B is preferred only for partial deprotonation of LAH4, specifically of His10 and His11, in accordance with the interpretation given in the original work.^43^ The alternative pattern of partial deprotonation corresponds to the opposite sign of ΔΔ*G*_AB_ and does not confirm a preference for this type of conformational transition. Finally, full deprotonation also does not favor the A → B transition, which is consistent with qualitative experimental data (there is no experimental structure for fully protonated LAH4). Moreover, the relatively low value of ΔΔ*G*_AB_ = 2.12 ± 2.04 kJ/mol for the case of full deprotonation implies a favored high-pH-compatible structure to be near state A, which is also in agreement with the experimental preferences of LAH4 for high pH values.

### C-Terminal Segment of Surfactant Protein B

In the case of the peptide fragment of the SPB protein, a change in the solvent composition leads to measurable structural changes, as demonstrated in ref (44). In hexafluoro-2-propanol, the five N-terminal residues of the peptide stay largely unstructured (state B), whereas in sodium dodecyl sulfate micelles, these residues adopt a well-defined compact conformation (state A). In our research, these two environments are represented by 2,2,2-trifluoroethanol and hexane, respectively. Based on the calculated relative free energy values, switching the solvent from hexane to 2,2,2-trifluoroethanol led to a reduction of the Δ*G*_AB_ value by −6.65 ± 0.04 kJ/mol relative to the reference system (state A, protein in hexane), indicating the significant role of the solvent. Furthermore, the sign of ΔΔ*G*_AB_ indicates that this change significantly shifts the conformational preferences toward state B. This result is in full agreement with the experimental data^44^ and the structures deposited in the PDB: 1RG3 and 1RG4, characteristic for nonpolar and moderately polar solvents, respectively.

### β_2_-Adrenergic Receptor

The two major types of β_2_-AR conformations and the equilibrium between them regulate the basis of its primary functions in living organisms, specifically signal transduction into the cell using G-proteins. These conformations can be classified into the inactive state, which is adopted in the presence of antagonists, inverse agonists, or in the absence of any ligand (state A),^45^ and the active state, which is induced by the presence of an agonist ligand, facilitating the binding of the receptor to G-proteins (state B).^46^ These conformations differ notably in the structure of the intracellular regions of the β_2_-AR, particularly in the arrangement of the sixth transmembrane (TM) domain relative to the other domains.^46^ It should be noted that the β_2_-AR receptor exhibits a measurable population of active conformers (approximately 0.5%) even in the absence of a bound ligand.^48^

The present study examined the effect of the lipid membrane composition in which the receptor is embedded on its conformational equilibrium. The transformation of the unliganded receptor from A to B under conditions of altered lipid membrane composition (100% DPPC → 100% POPC and 100% DPPC → 100% POPE) results in either an increase or a decrease in the Δ*G*_AB_ values relative to the reference system (state A, 100% DPPC). The relative changes in free energy are 10.01 ± 5.88 and −2.5 ± 4.09 kJ/mol, respectively. These values are significant compared to the estimated free energy required for the activation of unliganded β_2_-AR in real systems (ca. 13 kJ/mol),^48^ indicating a strong shift in the conformational equilibrium toward the active conformation. However, it is important to note that the modification in the lipid membrane composition examined in this study is extreme and involves 100% of the lipids contained in the membrane. Such alterations are unlikely to occur in realistic biomolecular systems, so the associated effect of membrane composition is much smaller.

Despite the absence of quantitative data regarding the impact of membrane composition on β_2_-AR activation, some qualitative data or findings concerning other G-protein-coupled receptors (GPCRs) exist, indicating the validity of the obtained results. From a purely qualitative perspective, refs (49) and (50) indicate that the content of unsaturated lipids in the membrane can affect the conformational equilibrium in GPCRs. Typically, higher levels of unsaturated components correlate with increased activity of either rhodopsin or adenosine A2A receptors. However, as indicated in ref (51) the effects of unsaturated lipids on receptor conformation may vary for different GPCRs, since many of the lipid-facing residues in the transmembrane (TM) regions are not highly conserved. Furthermore, the composition of the membranes considered is far from that of the homogeneous bilayers currently simulated. However, some effect is also expected for β_2_-AR due to the purely mechanical influence of increased lateral pressure^51^ that correlates with the content of unsaturated acyl chains in the bilayer. Reference (52) presents simulation results of the μ opioid receptor interacting with homogeneous bilayers. The study concludes that the maximum tilt angles of the fifth and sixth TMs (which are correlated with GPCR activation) are largest in the case of the DPPC bilayer compared to POPC. This is due to the variation in the membrane thickness and the associated movement of the helices, which keep them within the membrane. Such movement is compatible with the spatial expansion of the extracellular part of the receptor, characteristic for the activation process, and is expected to occur also in the case of β_2_-AR. Regarding the reverse trend in the ΔΔ*G*_AB_ values observed in for the POPE bilayer, there are several papers reporting on the influence of lipids capable of forming salt bridges with ionized residues in the extracellular part of β_2_-AR on its activation process.^53−56^ Interactions with lipid heads can disrupt certain essential interactions within the extracellular part of the GPCR, which promotes activation. A similar effect is also expected in the present case, since POPE is the phospholipid that, among the three compounds considered, has the greatest potential to form ionic bridges with protein due to the presence of ionized amine groups in the head part. Finally, it is worth noting that POPC and POPE support opposite trends in shifting the dynamic equilibrium of β_2_-AR activation compared to that of DPPC.

Another factor considered in the context of β_2_-AR was the presence of a ligand bound to the active site of the receptor. Propranolol and epinephrine act as antagonists and agonists, respectively. Their presence alters the dynamic equilibrium of the receptor to either the inactive state (state A) or the active state (state B), when compared with the receptor in the absence of a ligand. As expected, the presence of propranolol significantly increases the energy value of the A → B transformation, which quantitatively corresponds to the ΔΔ*G*_AB_ value of 12.7 ± 5.8 kJ/mol. However, very similar results were qualitatively and quantitatively obtained for epinephrine (ΔΔ*G*_AB_ = 12.4 ± 5.0 kJ/mol), in which case a different direction of the dynamic equilibrium shift was expected. This result can be explained by the limitations of the coarse-grained force field type, which, despite its ability to predict ligand binding sites, cannot capture subtle conformational changes at the molecular microswitch level. Such changes cause conformational changes in the intracellular part as a result of ligand–receptor interactions in the extracellular part.^57^ The presence of both ligands in the binding cavity results in similar ΔΔ*G*_AB_ values, indicating that, regardless of their actual pharmacological nature, they merely act as steric hindrances influencing the equilibrium between active and inactive states of β_2_-AR in the same direction.

For almost all systems for which conformational preferences were clearly determined by experimentally obtained structures, the predictions made by the method we used proved to be accurate. The only exception is the β_2_-AR+epinephrine system, where the discrepancy in conformational preferences is likely due to inaccuracies in the CG force field rather than inherent shortcomings in the proposed methodology (see the discussion above). Although a large part of the results obtained for β_2_-AR cannot be directly related to the experimental data, they are still interesting from the point of view of the possible influence of phospholipids on the conformational equilibrium of the receptor, which could be an additional factor complementing the recognized role of cholesterol.^58^

### GCN4 Leucine Zipper Protein

This system is unique compared to others for two reasons: first, state B is not explicitly defined by a specific structure (either taken from the PDB database or otherwise) but corresponds to the unfolded protein, i.e., by definition, to many different conformational states. Second, unlike previously discussed qualitatively estimated impacts of a given factor on the protein conformational change, in this case, there are quantitative experimental data that determine how introducing the point mutation Arg25Ala affects the free energy of protein folding. Calorimetric data related to the protein-in-water system^47^ show that such a mutation reduces the folding energy by 6.95 kJ/mol. However, both the native form and the mutant have the same conformation after folding, specifically a structure composed of a single helix stabilized by numerous ionic bridges (PDB: 2ZTA).

Current simulations predict a change in the free energy of folding corresponding to the aforementioned mutation of approximately 5.40 ± 2.62 kJ/mol, consistent in sign and differing by only ca. 1.5 kJ/mol from the experimental value. Moreover, it can be stated that the most significant impact on the ΔΔ*G*_AB_ value is the loss of electrostatic interaction between Arg25 (mutated residue) and Glu22, which residues form an ionic bridge in the case of state A. The average difference in electrostatic energies between the native protein and the mutant (both in state A) calculated based on CG MD trajectories is ca. 9.5 kJ/mol. Finally, it is worth noting that our results are close to those reported in ref (59) where, based on an AI algorithm, the change in folding energy caused by the Arg25Ala mutation was predicted to vary from −0.75 to 7.76 kJ/mol (depending on the method), with an average value of 4.88 kJ/mol.

### Influence of Force Constants

The impact of force constant values applied to the elastic network and perturbed during simulations was considered in the context of the SPB protein. As described in the Methods section, the two types of alteration were applied, namely, the force constants twice as large (i.e., mostly equal to 1000 kJ/mol/nm^2^) and twice as small (i.e., 250 kJ/mol/nm^2^) as in the original simulations. It should be noted that a 2-fold increase in the force constant still should provide adequate quantitative agreement with atomistic simulations according to ref (14). On the contrary, 2-fold smaller values are not recommended due to excessively loose protein structures compared to all-atom data. Such a large reduction in constants makes sense only for protein regions identified as disordered (which is the case for several regions in the systems considered in this study, including SPB).

The related results (not reported in Table 2) confirm that the ΔΔ*G*_AB_ value calculated for force constants twice as high as the original ones is close (−7.59 ± 0.19 kJ/mol) to the value obtained for the original setup, i.e., −6.65 ± 0.04. On the contrary, for the second set of modified force constants, the obtained ΔΔ*G*_AB_ value deviates more from the original, being equal to −1.40 ± 0.11 kJ/mol, which likely results from the enhanced flexibility of structures in limiting conformational states.

In summary, based on this case, it can be stated that changing the values of force constants has a small impact on the final ΔΔ*G*_AB_ values as long as the changes occur within the range of values, characteristic of the Martini force field.

### Other Remarks

The main advantages of the method presented here are

- The capacity to explore conformational transitions for protein systems with secondary and tertiary structures supported by restraints based on the elastic network approach, which is a standard approach in the case of the Martini force field.
- The ability to use the same, λ-based, limiting states A and B to study the influence of multiple external or internal factors influencing the dynamic equilibrium between them.
- High computational efficiency being a direct result of using the CG model.
- The implementation process is easy and requires only standard tools in MD simulation packages; it relies on a simple modification of topology files and calculating free energy based on the coupling parameter λ.
- It is easy to estimate both the magnitude of the simulation error and the convergence of the results.
- Possibility to extend its applicability to the case of other CG force fields which use analogous methodology to support the protein secondary and tertiary structure (i.e., elastic network approach or analogous, topology-based parameters) such as the SPICA force field.^60^

The most serious limitations of the proposed method include:

- The dependence on the knowledge of the structures defining the limiting conformational states A and B, which excludes the exploration of unknown conformations. In the absence of appropriate experimental information (e.g., structures based on spectroscopic or crystallographic studies), a substitute solution could be to use structures based on all-atom simulations or homology modeling.
- Operating with relative values of free energy changes, ΔΔ*G*_AB_. They are the main outcome, which indicates the direction of equilibrium state changes, but does not establish the absolute value of Δ*G*_AB_. In this case, some guidance on the significance of the relative free energy changes obtained can be provided by conditions corresponding to the boundary structures A and B.
- As demonstrated by the example of the β_2_-AR receptor, effects related to relatively subtle changes in ligand structure and their impact on the conformations of the respective molecular targets appear to be impossible to capture with the proposed approach. However, this conclusion is based solely on a single ligand-protein system. Addressing such a broad issue requires further research, which we plan to undertake in the near future.
- Technical difficulties in preparing correct files defining two conformations in the input parameter file (GROMACS topology). In this context, partial automation can be facilitated using the script provided in the Supporting Information.

## Conclusions

The study provides a computational protocol that estimates relative free energy changes resulting from conformational distortions in a protein induced by various factors (mutations, pH, solvent changes, etc.). The presented protocol is based on the Martini coarse-grained force field (version 3) and relies on the use of the coupling parameter λ, which drives conformational changes between two predefined conformational states. Subsequently, the free energy change associated with such a transition is computed by any method based on the λ formalism. The method was tested on five distinct biomolecular systems, where the conformational changes of proteins are induced by alterations in external or internal conditions experienced by the protein molecule. The proposed approach is straightforward and applicable within the standard simulation setup required for Martini-based CG MD simulations. It only requires knowledge of the two (or more) conformational states defining the transition of interest and the implementability of the factors affecting such a transition. The described approach can be particularly useful when considering a variety of factors acting on the same system and capable of disrupting the dynamic equilibrium between specific, well-defined protein conformations.

## Supporting Information Available

Bash script for generating a set of dual elastic networks (*mix_rubber_bands.sh*) and dual topology files (in GROMACS *itp format) for all proteins considered in this study are available free of charge via the Internet at http://pubs.acs.org (ZIP)
